# To the editor: comments on the paper: pressure monitoring devices may undetect epidural space: a report on the use of Compuflo® system for epidural injection

**DOI:** 10.1007/s10877-022-00868-4

**Published:** 2022-05-03

**Authors:** Mark Hochman, Giorgio Capogna

**Affiliations:** 1 Clinical Affairs, Research and Development, Milestone Scientific, Livingston, USA; 2 European School of Obstetric Anesthesia, Rome, Italy

To the editor,

In the issue of June Carassiti and other 6 collaborators [1] reported a case-series of epidural space misdetection by Compuflo system. All the cases were collected by the same anesthetist expert in epidural anesthesia who observed the 6 misdetection cases on a total number of 60 ambulatory epidural procedures for lumbar back pain with the Compuflo instrument.

CompuFlo is a computer-controlled drug delivery system that can precisely measure the pressure of human tissues in real-time at the orifice of a needle. It is capable of distinguishing different tissue types by providing continuously real-time “exit-pressure” data at the needle tip when placed in-situ. The instrument uses an algorithm to determine the pressure at the tip of the needle via a continuous fluid path. In this system the pressure is a feed-back loop and controller to the system, thus regulating the electro-mechanical motor which controls flow-rate and the fluid dispensed by the system. An audible and visual graphic of exit-pressure is provided to the health care practitioner enabling the operator to focus on the injection site. A sudden drop in pressure (typically greater than 50% of the maximum pressure) on the visual display accompanied by a distinct fall in the tone of the audio output, resulting in the formation of a low and stable pressure plateau sustained for more than 5 s, is considered consistent with the entry into the epidural space [2–5].

We believe that the criteria used to identify the epidural space in the case series reported by Carassiti et al. [1] led them to a misinterpretation of their data.

It is our opinion that if the authors had used the default settings suggested by the manufacturer, they would not have had the false negatives nor the uncertainty cases.

 Looking at their Fig. 1, we can conclusively see that the manufactory settings in the CompuFlo were changed from the default settings displayed on the bottom of the screen, and this can explain the findings reported by the Authors.

CompuFlo manufactory settings were changed from the default flow-rate of 0.050 mL/sec and high threshold pressure setting of 100 mmHg to a high pressure threshold setting of 200 mmHg and, more impactful, to a flow rate of 0.50 mL/sec.

The flow rate used by the Authors is 10 times greater than the default factory setting and more precisely is the maximum flow rate possible for the instrument.

These two changes clearly have affected the results.

In the figure reported in their letter, there was a drop from 200 to 150 mm/Hg (the maximum threshold pressure was set at 200 mm/Hg, and each horizontal line on the screen represents 50 mmHg). If the factory setting had been properly set at 100 mm/Hg, a drop of 50 mm/Hg would represent a 50% drop in pressure indicating the entry in the epidural space.

More importantly, the manufactory default flow rate to 0.050 mL/sec would have produced a much larger drop in pressure drop. With the flow rate set to 0.50 mL/sec, which is 10 times the factory default, once the need penetrates the ligamentum flavum and into the epidural space the tissues and fluid within the space create resistance to the flow out of the needle. Using such an extreme maximum flow-rate would produce resistances, i.e., back-pressure, and create an positive off-set to the pressure reducing the over percentage drop in pressure. That off-set produced by using the maximum flow-rate reduces a drop in pressure and that affects the acoustic pith of the tone. It is surprising that “*after a 1-month training on manikins*”, as reported by the Authors in their letter, this setting related issue was not noted by the Authors.

The device’s user manual identifies particular default settings which were selected by the company as the default settings based on prior research. All previous published studies successfully and exclusively used the default settings [2–5]. The instrument is capable of having these settings changed but one should only make a change to the default settings if they are based upon a newly established rationale and understanding consistent with the use of the instrument.

In their conclusion the Authors claims for more investigations on adequate setting for CompuFlo: if they had simply used the manufacturer original settings, they would not have had difficulty in interpreting the pressure curves because the curves produced would have been clearer and more readable, and most likely they would not have observed “false negatives”. The low percentage of pressure change due to unappropriated settings would also explain the lack of a distinct acoustic change reported.

In addition, by following the manufacture’s settings indications they would have used the instrument in a safer and more reliable way.

There are also a few other observations we’d like to make.

The first one is concerning the quotation of the Vaira’s study [4] which is misrepresented.

In their letter the Authors provided the following statement: “*Pressure monitoring devices have already been reported to give false negatives in detecting the epidural space by Carassiti et al.* [1]”, giving the reader the impression that other researchers have also reported misdetections of the epidural space. However, Vaira’s paper [4] did not studied “false-negatives” but demonstrated that the drop in pressure associated with the epidural space identification (true loss of resistance) was significantly greater than that recorded after the false loss of resistance. In addition, the *false negatives* reported by Vaira et. al. referred to the correct identification of the false loss of resistance due to the positioning of the epidural needle outside the epidural space and not to the CompuFlo’s failure to detect the epidural space.

The second observation is concerning the Authors declaration of absence of conflict of interest.

By reviewing the studies previously performed by this team it appears that they are developing a competing technology as declared by their own admission in one of their studies [6–9]. It is unfortunate that the researchers did not feel obligated to inform readers that they are actively researching and developing a different technology that is also based on a form of pressure sensing used for the epidural technique [9]. This omission could be misinterpreted since one could argue that they are potentially competitors to the instrument they have investigated and discussed.

## Data Availability

Not applicable.
